# Skeletal Maturity Might Not Be a Factor in Optimizing Outcomes in Total Hip Arthroplasty

**DOI:** 10.7759/cureus.77526

**Published:** 2025-01-16

**Authors:** Correggio L Peagler, Philip Parel, Kennedy Musgrave, Sarah Dance, Roxana Martinez, Savyasachi C Thakkar, Sean A Tabaie

**Affiliations:** 1 Department of Orthopaedic Surgery, George Washington University School of Medicine and Health Sciences, Washington, D.C., USA; 2 Department of Orthopaedic Surgery, Children’s National Hospital, Washington, D.C., USA; 3 Department of Orthopaedic Surgery, Case Western Reserve University Hospital, Cleveland, USA; 4 Department of Orthopaedic Surgery, Johns Hopkins Health System, Baltimore, USA; 5 Department of Orthopaedic Surgery, Nationwide Children’s Hospital, Columbus, USA

**Keywords:** pediatric orthopedic surgery, revision joint replacement, skeletal maturity, total hip arthroplasty: tha, total hip replacement (thr)

## Abstract

Introduction

Total hip arthroplasty (THA) is rarely indicated in the skeletally immature population. In these instances, there is concern for implant survival compared to the traditional older population. There has been a steady rise in the use of THA in the pediatric population due to improvements in surgical techniques. While the outcomes in THA for skeletally immature patients have been described in the literature, there are no population studies looking at this procedure in a skeletally immature individual. Therefore, the purpose of this study was to compare 10-year implant survivability following primary THA in skeletally mature versus skeletally immature patients.

Methods

Patients who underwent primary THA were identified using a large national database (PearlDiver). THA patients were then divided into presumed skeletally immature male patients (0-16 years), presumed skeletally mature male patients (17-21 years), presumed skeletally immature females (0-14 years), and presumed skeletally mature females (15-21 years). Multivariable analysis was conducted using Cox proportional hazards modeling to determine differences in the risk of revision for periprosthetic joint infection (PJI), mechanical loosening, dislocation/instability, and periprosthetic fracture (PPF).

Results

In total, 352 male patients (244 skeletally mature and 108 skeletally immature) and 409 female patients (350 skeletally mature and 59 skeletally immature) were identified. Compared to skeletally immature females, skeletally mature females had no higher risk of 10-year revision for all-cause, PJI, mechanical loosening, dislocation/instability, or PPF (P > 0.05 for all). Compared to skeletally immature males, skeletally mature males had no higher risk of 10-year revision for all-cause, PJI, mechanical loosening, dislocation/instability, or PPF (P > 0.05 for all).

Conclusion

Although THA occurred more commonly in patients above the presumed age of skeletal maturity, the lack of significantly different surgical outcomes suggests that younger age and presumed skeletal immaturity may not put patients at any further risk of implant failure. While further research is needed to understand the impact of age and skeletal maturity on outcomes of THA, these results indicate that the initial age of a THA may not be a factor in optimizing outcomes, and suggests that orthopedic surgeons need not delay surgery based on age or skeletal maturity alone.

## Introduction

Total hip arthroplasty (THA) is a revolutionary surgical procedure that has been widely indicated in the relief of chronic hip pain due to arthritis. Since its inception, technical improvements have increased the efficacy of THA, making it the leading solution for degenerative joint arthritis in adults and geriatric patients [1,2]. Despite its success in these populations, it has been used less frequently in the pediatric population. The complexities associated with the surgery-as it relates to skeletal immaturity-often persuade physicians to take alternative routes when treating pediatric patients with end-stage joint disease [3,4].

The pediatric population is unique in that degenerative joint disease is fairly uncommon and the etiology of hip disease is usually indicative of more complicated disease processes, such as malignancy, sickle cell disease, Legg-Calve-Perthes, and congenital hip deformity [5-9]. In this population, many interventions, such as dega osteotomy and varus derotation osteotomy, are employed prior to THA. The increased risk of failure of these operations is attributed to bilateral hip involvement and advanced-stage avascular necrosis [10]. Some literature demonstrates that pediatric patients are at an increased risk for post-surgical complications, such as implant failure, due to large leg-length discrepancies and poor implant survivorship due to age and high activity levels in young patients, leading to THA being a last resort [4].

To date, there is limited literature on THA in skeletally immature patients, with most studies coming from single institutions and having small (<20) cohorts of patients [3,4,11]. The purpose of this study is to analyze THA outcomes in presumed skeletally immature patients compared to their skeletally mature counterparts.

This study was also previously presented as a poster at the 19th Annual International Pediatric Orthopaedic Symposium, December 5-9, 2023.

## Materials and methods

Database

A retrospective cohort analysis was performed using the Mariner dataset from the PearlDiver Patient Records database (www.pearldiverinc.com), a database accessible to healthcare professionals, institutions, and researchers through subscription. The Mariner dataset contains all payers’ claims information of more than 157 million patients from 2010 to 2021. Mariner provides longitudinal follow-up data utilizing unique patient identifier codes that are not limited to changes in insurance status, thus minimizing loss to follow-up in the system. Additionally, it provides only de-identified information to users, exempting this study from Institutional Review Board approval.

Patient population

Patients who underwent primary THA were identified using Current Procedural Terminology (CPT) and International Classification of Diseases (ICD-10) procedure codes. Patients were included if they underwent THA with at least a 2-year follow-up and were followed for a maximum of 10 years. Patients who underwent a contralateral THA within two years of the first procedure were excluded to control for the laterality of the surgical outcomes. Following this criteria, patients were stratified into “presumed skeletally mature” and “presumed skeletally immature” by sex using an age of 16 and 14 as the cutoff for maturity for males and females, respectively, based on prior literature [12,13]. Patients over the age of 21 were excluded.

Demographic, comorbidities, and outcome variables

Demographic characteristics included age, gender, and the Charlson Comorbidity Index (CCI). The primary outcome was the 10-year cumulative incidence for all-cause revision. Secondary outcomes included indications for revisions in presumed skeletally mature and immature patients. Observed indications included periprosthetic joint infection (PJI), mechanical loosening, dislocation and instability, and periprosthetic fracture (PPF).

Statistical analysis

Patient demographics, 10-year surgical outcomes, and indications for 10-year surgical outcomes between presumed skeletally mature and immature patients were conducted using univariate analysis. Demographics and indications were analyzed using the chi-square and student's t-test where appropriate. These results were recorded as the number of patients in each category, the incidence/prevalence rate (%), and the p-value. The Cox proportional hazard model was utilized to observe significant differences in the cumulative incidence rates with the output recorded as the hazard ratios (HR), 95% confidence intervals (95% CI), and the p-value. A p-value less than 0.05 was used as the significance level for this study. All statistical analysis was conducted using R software (Vienna, Austria) provided by the PearlDiver database.

## Results

Demographics and clinical comorbidities

In total, 419 male patients and 471 female patients were included in this study, with 289 (69%) and 395 (84%) patients classified as presumed skeletally mature, respectively. Demographics and clinical comorbidity data can be found in Table 1.

**Table 1:** Demographic and clinical comorbidities of pediatric patients A student's t-test was performed to assess for statistical significance. P-value <0.05 was considered statistically significant.

	Skeletally Mature Cohort		Skeletally Immature Cohort		
	Mean	Standard Deviation	Mean	Standard Deviation	p-value
Male Patients					
Total (N)	289 (68.9%)	-	130 (31.02%)	-	-
Age (Years)	19.10	1.42	13.61	3.28	<0.001
Charlson Comorbidity Index	1.31	2.01	0.98	1.87	0.105
Female Patients					
Total (N)	395 (83.9%)	-	76 (16.1%)	-	-
Age (Years)	18.71	1.93	11.74	3.51	<0.001
Charlson Comorbidity Index	1.12	1.63	0.83	1.18	0.144

Cumulative incidence of revision

Compared to presumed skeletally immature patients, presumed skeletally mature patients had no higher risk of all-cause revision or etiologies for revision, including PJI, mechanical loosening, dislocation and instability, and periprosthetic fracture (P > 0.05 for all; Table 2) in males and females.

**Table 2:** Cumulative incidence and risk of 10-year revision by cause of revision in pediatric male patients: skeletally mature versus immature patients *PJI = Periprosthetic Joint Infection; PPF = Periprosthetic Fracture *Hazards ratio compares skeletally mature to skeletally immature patients.

	Hazards Ratio	95% Confidence Interval	P-Value
Male Patients			
All-Cause Revision	2.19	0.48-10.02	0.311
PJI	0	0-inf	0.999
Mechanical Loosening	1.32	0.26-6.56	0.731
Dislocation Instability	0.45	0.03-7.16	0.570
PPF	0	0-inf	0.999
Female Patients			
All-Cause Revision	1.35	0.17-10.74	0.773
PJI	0	0-inf	0.999
Mechanical Loosening	0	0-inf	0.999
Dislocation Instability	0.68	0.08-5.85	0.726
PPF	0	0-inf	0.999

## Discussion

With steadily increasing rates of THA in the pediatric population, understanding the impact THA has on the developing musculoskeletal system is paramount [9,14]. Moreover, further exploration of the impact of THA on skeletally immature individuals provides insight into an intervention timeline for pediatric hip diseases. Our study sought to determine if there was an increased risk of all-cause revision or etiologies for revision, including PJI, mechanical loosening, dislocation and instability, and periprosthetic fracture, in presumed skeletally immature patients compared to presumed skeletally mature patients. We found that in both presumed skeletally immature males and females, there was no increased risk of all-cause revision or etiologies for revision for either sex group.

As patients in current literature undergoing THA with a skeletally immature hip are defined as having open triradiate cartilage, we hypothesized that presumed skeletally immature patients would have an increased risk of all-cause revision or etiologies for revision, including PJI, mechanical loosening, dislocation and instability, and periprosthetic fracture. Current literature acknowledges previous beliefs of skeletal maturity’s impact on the outcome of THA was not evidence-based. Some theories in the literature as to why this was a common belief is due to increased wear and tear of implants, increased patient activity level, and growth potential of immature bones [14]. Interestingly, we found that compared to skeletally immature patients, skeletally mature patients had no higher risk of all-cause revision or etiologies for revision, including PJI, mechanical loosening, dislocation and instability, and periprosthetic fracture (all P > 0.05). These findings parallel previous research by Rainer et al. who found that, of the 12 patients (13 hips) with radiographically confirmed skeletal immaturity, only one patient experienced acetabular component loosening and instability [4]. While the population size of the previous study was not enough to guide surgical decisions, the majority of positive outcomes encouraged our study design of using the national claims database.

Current literature highlights the effectiveness of THA in pediatric patients despite the unique challenges that young age, increased physical activity, and distinct disease processes provide. Van de Velde et al. reported remarkably improved gait in 18 patients (16 female, 2 male) under the age of 16 undergoing THA with an average 3.8 years follow-up; an adductor tenotomy due to a persistent contracture was the only complication. Furthermore, four wheelchair-bound patients improved to ambulating without assistive devices following THA [11]. Additionally, several population studies have noted a steadily increasing trend for the use of THA to treat pediatric hip diseases such as osteonecrosis, congenital hip deformities, and Legg-Calve Perthes Disease [5-9]. The aforementioned conditions cause major decreases in the quality of life in the pediatric patient such as debilitating pain caused by necrosis of the femoral head and impaired ambulation caused by anatomical abnormalities of the acetabulum. While there is limited research surrounding the use of THA in the presumed/radiographically confirmed skeletally immature individual, the few single-institution studies that have examined this population have seen positive results [3,4,11]. Our study adds to the conversation by providing data from a nationally representative claims database.

While this study uses information from an all-payer national database that allows for the observation of long-term complications, it is not without limitations. The database relies on ICD and CPT codes where patients may be misclassified. Data results may be impacted by miscoding even though the database is highly regulated for validity and reliability. In terms of sample size, patients in the presumed skeletally mature accounted for 80% of the entire population making it challenging to make definitive conclusions from the data. This study was not able to radiographically confirm skeletal immaturity or other chart-specific information, such as leg length discrepancy, surgical approach, and patient-reported hip scores, due to its reliance on CPT and ICD codes. Therefore, we are unable to make definitive conclusions about skeletal immaturity’s impact on THA outcomes and can only base our claims on presumed skeletal immaturity. In future research, it would be valuable to radiographically confirm skeletal maturity in a population size more representative of the national population. Additionally, further research comparing the effectiveness of alternative interventions, such as osteotomy, to THA would further validate our claims.

## Conclusions

The present study compared the all-cause revision rate between skeletally immature and mature patients following THA, finding no higher risk for revision in both the male and female cohorts. To our knowledge, this is the first study to do so on a national level spanning over a 10-year period. Larger, longer-term, and controlled research is required to better determine the role of THA in skeletally immature patients with end-stage hip disease.
